# Ataxia induced by a thymic neuroblastoma in the elderly patient

**DOI:** 10.1186/s12957-015-0594-6

**Published:** 2015-05-12

**Authors:** Ory Wiesel, Shamik Bhattacharyya, Henrikas Vaitkevicius, Sashank Prasad, Ciaran McNamee

**Affiliations:** Division of Thoracic Surgery, Brigham and Women’s Hospital, Harvard Medical School, 75th Francis Street, Boston, 02115 MA USA; Division of Neurology, Brigham and Women’s Hospital, Harvard Medical School, 75th Francis Street, Boston, 02115 MA USA

**Keywords:** Neuroblastoma, Ataxia, Opsoclonus, Thymus, Anterior mediastinal mass

## Abstract

Thymic neuroblastoma is a rare tumor with only few reports in modern literature. Whereas most data is taken from childhood neuroblastoma, little is known about the characteristics of the disease in the adult and elderly population.

There are significant differences between adult and childhood neuroblastoma which are reviewed below.

We report a case of a 62-year-old male who presented with neurological symptoms of ataxia and opsoclonus and an anterior mediastinal mass. Ultimately, the patient underwent a resection of the mass and pathologic review identified a thymic neuroblastoma. This is the first case of thymic neuroblastoma associated with symptomatic central nervous system disease; it is presented with an up-to-date review of the previous cases in the field as well with a review of the literature of post adolescent neuroblastoma.

## Background

Neuroblastoma is a tumor originating from the neural-crest cell, arising from the sympathetic nervous system. Most of the reported cases arise in infants and children below the age of 10 years. In the adult and elderly population, it is a rare tumor.

While neurological symptoms may be associated in adults with thymic mass (as in myasthenia gravis), thymic neuroblastoma was never described as a cause for neurological symptoms in the adult population.

We present a case of a 62-years-old male who presented with neurological symptoms and was found to have thymic neuroblastoma. After a comprehensive workup, surgical resection with complete removal of the tumor was done. The patient recovered well, and his neurological symptoms subsided. Up to date, review of the current literature is presented with suggested workup and treatment plan based on the current limited literature.

## Case presentation

**Figure 1:**
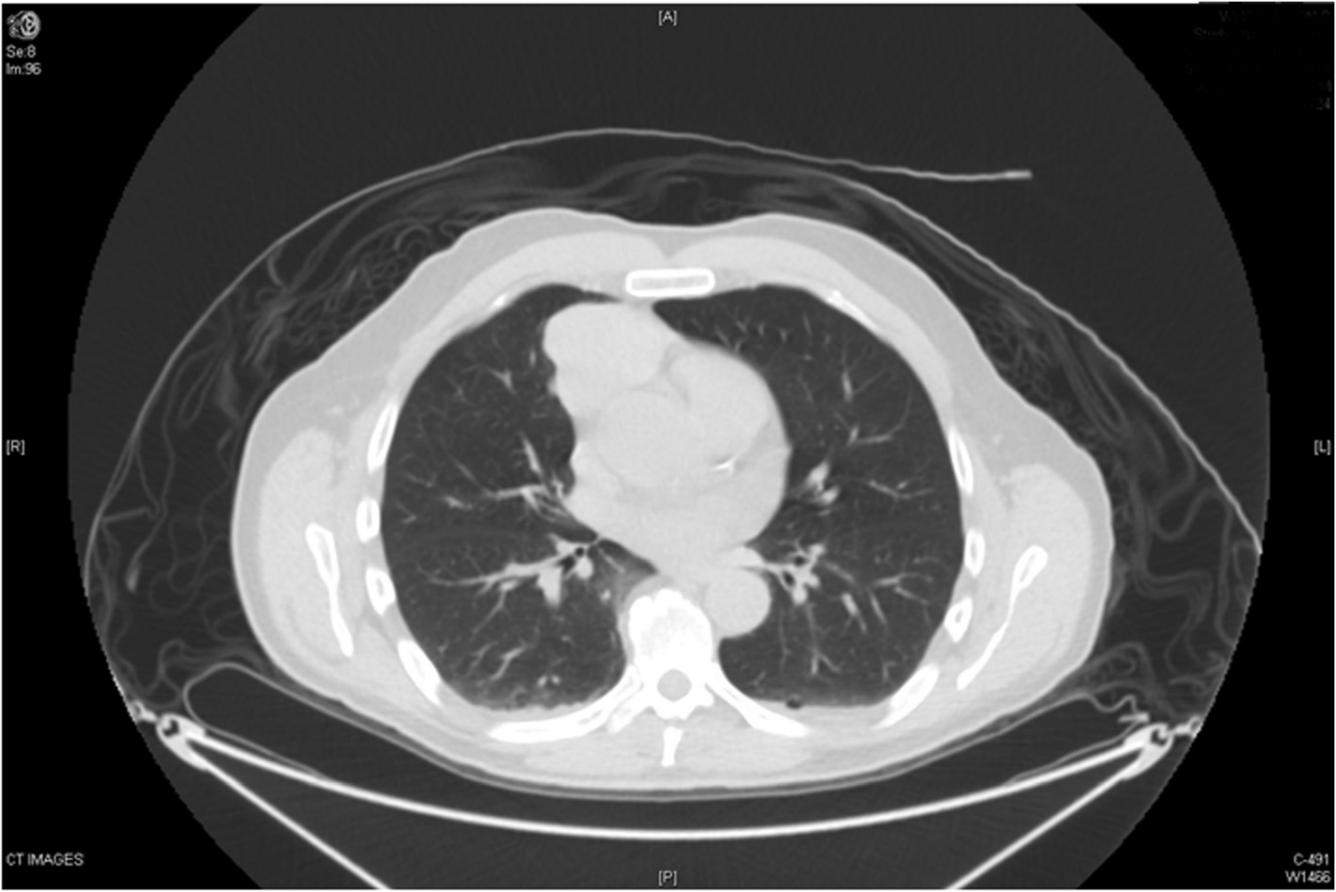
Axial section of initial CT scan with the anterior mediastinal mass abutting the pericardium.

**Figure 2:**
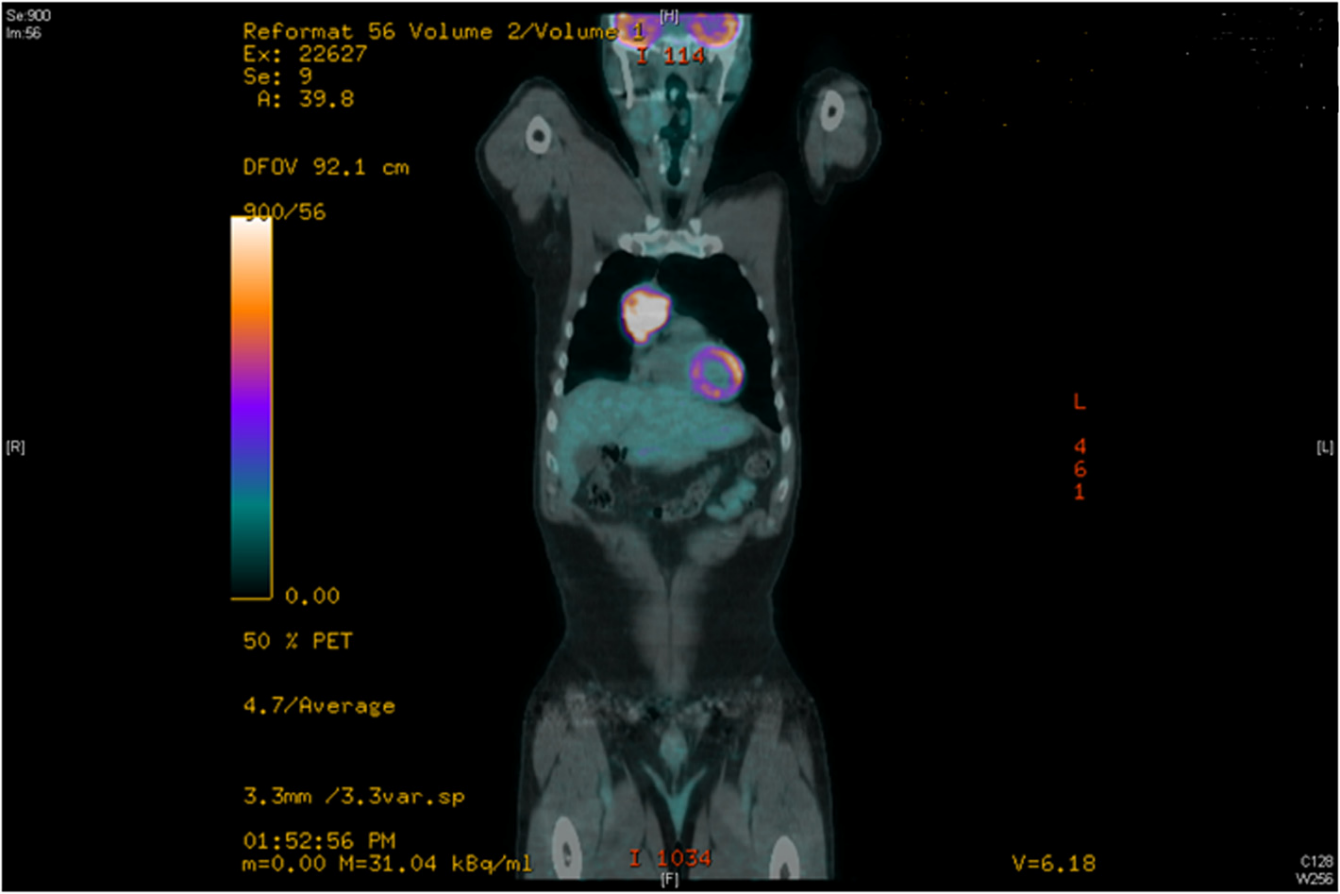
Positron emission tomography (PET) CT with intensely hypermetabolic 5.2 × 3.8 cm homogeneous anterior mediastinal mass (SUV max 15.94).

A previously asymptomatic 62-year-old male presented with ataxia and oscillopsia with progression over a 6-week interval. On physical examination, the patient was noted to have horizontal and vertical opsoclonus, truncal ataxia, right eye ptosis, and a hoarse voice. A chest CT was done and an anterior mediastinal mass was found (Figure 1). A magnetic resonance imaging (MRI) of the mediastinum revealed an anterior mediastinal mass extending from the distal ascending aorta to the right ventricular free wall with a smaller adjacent nodule, both abutting but not invading the neighboring structures. The mass was hyperintense on T1 and T2, with avid enhancement during first pass perfusion and heterogeneous enhancement on delayed images. Positron emission tomography (PET)/CT revealed a 5.2 × 3.8 cm anterior mediastinal mass with a superior satellite nodule (1.8 × 1.5 cm); both showed intense FDG avidity (Figure 2).

Further investigations included a brain MRI which revealed only chronic microvascular changes with an old lacunar infarction. Electromyography (EMG) including eye musculature did not show decrement in muscle activity with repetitive stimulation and was not consistent with myasthenia gravis. A spinal tap did not reveal malignant cells or oligoclonal bands.

The patient was commenced on treatment for a paraneoplastic cerebellar syndrome with intravenous immune globulin which provided no further improvement in his symptoms. Investigations previously yielded a negative serum panel for myasthenia gravis and serum alpha-fetoprotein (AFP) and beta-human chorionic gonadotropin (beta-hCG) serum levels were within normal limits; the patient’s serum and urine studies did not suggest syndrome of inappropriate anti diuretic hormone secretion (SIADH).

Following these investigations, the patient underwent a trans-sternal thymectomy, with adjacent pericardiectomy. The mass was dissected free from the superior vena cava and innominate vein without evidence of invasion of these structures. Three months after his surgery, the patient subjectively reported a slow progressive improvement in his neurologic symptoms and was able to walk independently with mostly resolved ocular flutter and mild dysarthria. Unfortunately, his ataxia and dysarthria became worse 6 months following surgery. Metastatic workup yield no evidence of recurrent disease. The patient was treated with chest irradiation and systemic immunosuppression with progressive improvement.

**Figure 3:**
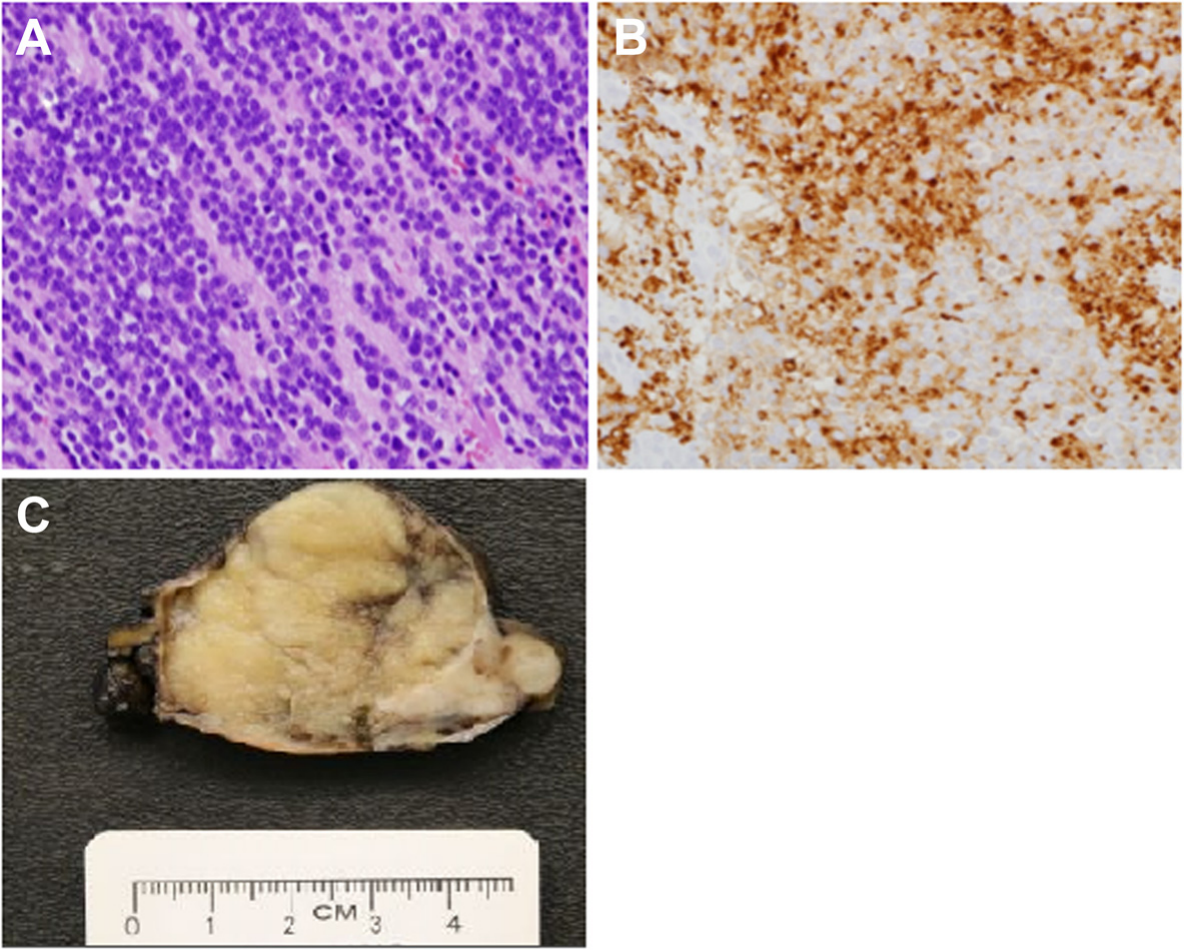
Histology, immunohistochemistry, and gross pathology of the resected specimen. **(A)** Histology of the mediastinal mass revealed a monotonous population of small- to medium-sized round cells with scant cytoplasm and finely granular chromatin within a background of neutrophils, consistent with the diagnosis of neuroblastoma (H&E, original magnification ×400). **(B)** Confirmatory immunohistochemistry (IHC) revealed diffuse synaptophysin positivity (original magnification ×400). **(C)** Pathologic specimen shows 7.5 × 5 × 3.7 cm, tan-white, lobulated, focally hemorrhagic, encapsulated resected anterior mediastinal mass.

Pathology revealed a 7.5-cm neuroblastoma without capsular invasion. The tumor was adherent to the pericardium but without invasion of neighboring structures including the pericardium. Resection margins were negative for tumor. One removed lymph node was negative for tumor. Immunohistochemistry staining was positive for synaptophysin, chromogranin, and NSE and negative for AE1, AE3, and CD99 (Figure 3). Tumor sequencing revealed no amplification of n-myc oncogene.

## Discussion

Neuroblastoma is a tumor originating from neural crest cell, arising from the sympathetic nervous system. Greater than 90% of the reported cases arise in infants and children below the age of 10 years, in whom it is known to be the most common extra-cranial solid tumor [1]. In the adult population, it is a rare tumor (0.2 new cases per million inhabitants per year [2]), whereas in the elderly population, it is extremely rare.

In a large surveillance, epidemiology, and end results (SEER) database between 1973 and 2007, 3,818 neuroblastoma patients (3,602 pediatric, 181 adults (28-60 years), and 35 elderly patients (>60 years)) were identified with this disease. The most common primary site for the elderly population was the heart and soft tissue (60%), respiratory system and mediastinum as a group (31.4%), and testis (5.7%). Only 2 of the 3,818 patients over the 30 years of data acquisition were found to have a primary mediastinal tumor site [3].

Thymic neuroblastoma is common in children less than 18 years of age; 42.9% of childhood neuroblastoma originates in the thymus and endocrine glands [3]. Adult onset thymic neuroblastoma has only been reported anecdotally in the modern literature and is limited to a few case reports which will be reviewed below [3-9].

Mortality rate is unknown for the elderly population. Most reports define follow-up after surgery as an end point, and they do not define standard disease-free intervals or mortality data. The survival rates observed for a group of 125 adults (age >20 years) with neuroblastoma in all locations was 45.9% and 36.3% for 3 and 5 years, respectively. A third of the patients had metastatic disease on presentation and they had lower survival rates than that of infants, children, and adolescents. Furthermore, long-term survival of adult patients has revealed a continuous decrease in survival over the first 7 years of follow-up with stabilization thereafter to a 33.3% survival at 10 years [2].

Reviewing the modern literature (1966-2014), for adult onset neuroblastoma, we found only nine reports (not including our patient) of elderly patients (>60 years) with primary mediastinal neuroblastoma; five of these were reported in the thymus, three cases in the anterior mediastinum, and one case in the superior mediastinum (Table 1).

**Table 1:** **Up-to-date review of the current literature - primary mediastinal neuroblastoma**

**Patient number**	**Primary site**	**Age/sex**	**Treatment**	**Size**	**Presenting symptom**	**n-myc amplified**	**Metastases**	**Outcome**	**Ref**
1	Left anterior mediastinum - thymus	65/F	Surgery alone	6.4 × 6.2 × 3.4 cm	None (abnormal CXR)	NA	None	NED at 15 months	Ueda et al. [9]
2	Left anterior mediastinum - thymus	79/F	Surgery alone	10 × 8 × 7 cm	SIADH, abnormal CXR	NA	None	NED at 24 months	Pellegrino et al. [7]
3	Thymus	67/F	CABG + resection	20 g	Coronary artery disease, SIADH	NA	NA	Died from VF POD 10	Argani et al. [5]
4	Anterior mediastinal mass	80/M	Surgery alone	7.3 cm	NA	NA	NA	NED at 18 months	Argani et al. [5]
5	Anterior mediastinal mass	71/F	Surgery alone	630 g	Abnormal CXR + pleural effusion	NA	Local + distant	Died 1 year from extensive disease	Argani et al. [5]
6	Superior mediastinum	64/M	Surgery + chemotherapy	5 × 3 cm	None (abnormal CXR)	+	Lymph node	Lymph node metastases at 10 months	Ohtaki et al. [4]
7	Right anterior mediastinum - thymus	60/M	Surgery alone	4.7 cm	SIADH, Abnormal CXR	NA	None	NA	Ogawa et al. [6]
8	Anterior mediastinum	80/F	Surgery + radiation therapy	7 cm	SIADH	NA	None	NED at 14 months	Salter et al. [8]
9	Left anterior mediastinum	86/M	Surgery alone	5.2 × 4.7 cm	SIADH, abnormal CXR	NA	None	NED at 11 months	Rogowitz et al. [3]
10	Anterior mediastinum - thymus	62/M	Surgery alone	5.2 × 3.8 cm	Ataxia, visual disturbances	Negative	None	NED at 6 months	Present report

CABG, coronary artery bypass graft; CXR, chest X-ray; VF, ventricular fibrillation; NED, no evidence of disease; SIADH, syndrome of inappropriate anti diuretic hormone secretion.

### Presumed pathogenesis of mediastinal neuroblastoma

The origin of thymic neuroblastoma is currently unknown but is thought to arise from four possible origins [5]:

1. Aberrantly located sympathetic ganglia cells in the thymus
2. Neuroectodermal cells residing in the normal thymus
3. Precursors of thymic epithelial cells with potential to differentiate along neuronal lines
4. Malignant transformation of mediastinal teratomas

#### Clinical presentation

Neuroblastoma in childhood may be associated with neurologic symptoms such as opsoclonus-myoclonus syndrome (OMS) attributable to immune-mediated injury of the cerebellum and likely other targets within the central nervous system. The syndrome manifests itself as opsoclonus, myoclonic jerks, ataxia, and/or encephalopathy. A search of the literature reveals no reports of adult patient with neuroblastoma and OMS [10,11].

There are scattered case reports in adults of mediastinal neuroblastoma and increased secretion of anti diuretic hormone (ADH) [5-7]. SIADH is known to arise from neuroendocrine cells and is associated with paraneoplastic syndromes with several thoracic malignancies: small cell lung cancer and thymic neoplasms (thymoma, thymic carcinoma, thymic ganglioneuroblastoma).

As compared to childhood neuroblastoma, adult onset neuroblastoma has a worse outcome independent of stage, primary site, and clinical course on presentation. This is possibly due to chemotherapy which may be less active in adults than in children [1]. Clinical data show that even loco-regional neuroblastoma, which is considered curable in children with minimal therapy, has a poor outcome for adolescents and adults. Adult neuroblastoma patients suffer from multiple recurrences, with poor outcomes with recurrence.

Neuroblastoma occurring in children may express n-myc oncogene which is a sign of aggressive disease [2], and some express catecholamines. Adult neuroblastoma rarely expresses n-myc amplification and only a few secrete catecholamines [1].

Given the rare incidence, the natural history of elderly (>60 years) thymic neuroblastoma is unknown. In childhood neuroblastoma, prognosis is inversely related to the age of presentation (infants carry the best prognosis); there is a consensus that in adults, the disease is more indolent but carries a worse prognosis. However, mediastinal neuroblastoma carries a better prognosis in the elderly compared to the other groups [1].

Surgery with or without chemotherapy is the mainstay of treatment for neuroblastoma in childhood (radiotherapy is reserved for tumor progression). However, in the adult population, given the rarity of the disease and limited data, there are no standard treatment guidelines and most pediatric protocols are not tolerated by the adult population. Expert opinion suggests surgical resection with negative surgical margins as the main treatment and adjuvant chemotherapy or radiotherapy for advanced disease [3].

This is the first report of elderly patient with thymic neuroblastoma presenting with positive neurological symptoms which improved following resection of the tumor.

## Conclusions

Thymic masses presenting in the elderly should be thoroughly investigated and in the absence of metastatic disease should be resected with negative margins if possible. Neuroblastoma should be considered in the differential diagnosis of thymic mass with SIADH-related syndrome or nonmyasthenic neurological symptoms. Surgery with negative margins for adult neuroblastoma is recommended; however, recurrence may occur.

## Consent

Written informed consent was obtained from the patient for publication of the case report and any accompanying images. A copy of the written consent is available for review by the editor in chief of this journal.

## Abbreviations

ADH: anti diuretic hormone
Beta HCG: beta human chorionic gonadotropin
CABG: coronary artery bypass graft
CXR: chest X-ray
EMG: electromyography
MRI: magnetic resonance imaging
NED: no evidence of disease
OMS: opsoclonus myoclonus syndrome
PET: positron emission tomography
SEER: surveillance, epidemiology, and end results
SIADH: syndrome of inappropriate anti diuretic hormone secretion
VF: ventricular fibrillation

## References

[1] Kushner BH, Kramer K, LaQuaglia MP, Modak S, Cheung NK (2003). Neuroblastoma in adolescents and adults: the Memorial Sloan-Kettering experience. Med Pediatr Oncol.

[2] Esiashvili N, Goodman M, Ward K, Marcus RB, Johnstone PA (2007). Neuroblastoma in adults: incidence and survival analysis based on SEER data. Pediatr Blood Cancer.

[3] Rogowitz E, Babiker HM, Kanaan M, Millius RA, Ringenberg QS, Bishop M (2014). Neuroblastoma of the elderly, an oncologist's nightmare: case presentation, literature review and SEER database analysis. Exp Hematol Oncol.

[4] Ohtaki Y, Ishii G, Hasegawa T, Nagai K (2011). Adult neuroblastoma arising in the superior mediastinum. Interact Cardiovasc Thorac Surg.

[5] Argani P, Erlandson RA, Rosai J (1997). Thymic neuroblastoma in adults: report of three cases with special emphasis on its association with the syndrome of inappropriate secretion of antidiuretic hormone. Am J Clin Pathol.

[6] Ogawa F, Amano H, Iyoda A, Satoh Y (2009). Thymic neuroblastoma with the syndrome of inappropriate secretion of antidiuretic hormone. Interact Cardiovasc Thorac Surg.

[7] Pellegrino M, Gianotti L, Cassibba S, Brizio R, Terzi A, Borretta G. Neuroblastoma in the elderly and SIADH: case report and review of the literature. Case Rep Med. 2012. doi:10.1155/2012/952645 10.1155/2012/952645 PMC3432386 22956963

[8] Salter JE, Gibson N, Mackay B (1995). Neuroblastoma of the anterior mediastinum in an 80-year-old woman. Ultrastruct Pathol.

[9] Ueda Y, Yuba Y (2012). Thymic neuroblastoma within a thymic cyst in an adult. Case Rep Oncol.

[10] Jasminekalyani P, Saravanan S.Dancing eyes dancing feet syndrome-a report of two cases.J Clin Diagn Res. 2014;8:MD03–05 10.7860/JCDR/2014/7184.4339 PMC4080025 24995204

[11] Matthay KK, Blaes F, Hero B, Plantaz D, De Alarcon P, Mitchell WG (2005). Opsoclonus myoclonus syndrome in neuroblastoma a report from a workshop on the dancing eyes syndrome at the advances in neuroblastoma meeting in Genoa, Italy, 2004. Cancer Lett.

